# Poly[[(μ_2_-4,4′-bipyridyl-κ^2^ *N*:*N*′)bis­{μ_2_-*N*-[2-(2-hy­droxy­benzo­yl)carbamo­thio­yl]acetamidato-κ^4^ *O*,*N*,*O*′:*S*}bis­(nitrato-κ^2^ *O*,*O*′)dicadmium] di­methyl­formamide tetra­solvate]

**DOI:** 10.1107/S1600536813030055

**Published:** 2013-11-09

**Authors:** Ming-Hua You, Fo-Jun Li, Xiao-Ping Yang, Xu-Xiang Lin, Hua-Yan Ye

**Affiliations:** a Zhicheng College, Fuzhou University, Fuzhou, Fujian 350108, People’s Republic of China

## Abstract

The asymmetric unit of the title complex, {[Cd_2_(C_10_H_10_N_3_O_3_S)_2_(C_10_H_8_N_2_)(NO_3_)_2_]·4C_3_H_7_NO}_*n*_, consists of one Cd^II^ cation, one *N*-[2-(2-hy­droxy­benzo­yl)carbamo­thio­yl]acetamidate ligand, half a 4,4′-bipyridyl ligand, one coordinating nitrate anion and two di­methyl­formamide solvent mol­ecules of crystallization. The bipyridine ligand is completed by inversion symmetry. The metal cation exhibits a distorted penta­gonal–bipyramidal coordination geometry provided by two O and one N atoms of the thio­semicarbazide ligand, two O atoms of the nitrate anion, one S atom of a neighbouring thio­semicarbazide ligand and one 4,4′-bi­pyridine N atom. The bridging role of the thio­semicarbazide ligand through the S atom leads to centrosymmetric binuclear units, which are further linked by 4,4′-bi­pyridine units, forming polymeric chains extending along the *b-*axis direction. An intra­molecular N—H⋯O hydrogen bond occurs. The crystal structure also features N—H⋯O and O—H⋯O hydrogen bonds, leading to the formation of a three-dimensional network.

## Related literature

For background to the properties and applications of thio­semicarbazone complexes, see: Quiroga & Ranninger (2004 ▶); Kasuga *et al.* (2003 ▶); Floquet *et al.* (2009 ▶); Hassanien *et al.* (2008 ▶); Latheef *et al.* (2006 ▶); Babb *et al.* (2003 ▶). For related structures, see: Ke *et al.* (2007 ▶); Wang *et al.* (2010 ▶); Liu *et al.* (2013 ▶). For the synthesis of the *N*-(2-(2-hy­droxy­benzo­yl)carbamo­thio­yl)acetamide ligand, see: Wang *et al.* (2000 ▶). 
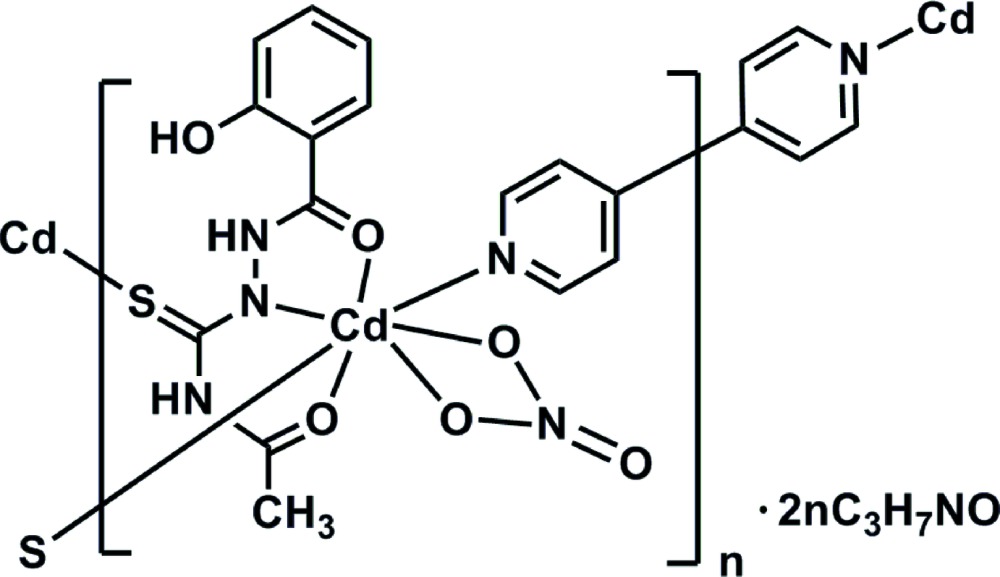


## Experimental

### 

#### Crystal data

[Cd_2_(C_10_H_10_N_3_O_3_S)_2_(C_10_H_8_N_2_)(NO_3_)_2_]·4C_3_H_7_NO
*M* *_r_* = 1301.93
Monoclinic, 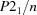

*a* = 13.831 (3) Å
*b* = 15.280 (3) Å
*c* = 14.363 (3) Å
β = 110.55 (3)°
*V* = 2842.4 (12) Å^3^
*Z* = 2
Mo *K*α radiation
μ = 0.90 mm^−1^
*T* = 293 K
0.30 × 0.18 × 0.08 mm

#### Data collection

Rigaku Saturn 724+ CCD diffractometer
Absorption correction: numerical (*CrystalClear*; Rigaku, 2007 ▶) *T*_min_ = 0.813, *T*_max_ = 0.946
18197 measured reflections
4980 independent reflections
4753 reflections with *I* > 2σ(*I*)
*R*_int_ = 0.043

#### Refinement

*R*[*F* ^2^ > 2σ(*F* ^2^)] = 0.060
*wR*(*F* ^2^) = 0.141
*S* = 1.28
4980 reflections
349 parameters
H-atom parameters constrained
Δρ_max_ = 0.70 e Å^−3^
Δρ_min_ = −0.63 e Å^−3^

### 

Data collection: *CrystalClear* (Rigaku, 2007 ▶); cell refinement: *CrystalClear*; data reduction: *CrystalClear*; program(s) used to solve structure: *SHELXS97* (Sheldrick, 2008 ▶); program(s) used to refine structure: *SHELXL97* (Sheldrick, 2008 ▶); molecular graphics: *SHELXTL* (Sheldrick, 2008 ▶); software used to prepare material for publication: *SHELXL97*.

## Supplementary Material

Crystal structure: contains datablock(s) I, New_Global_Publ_Block. DOI: 10.1107/S1600536813030055/rz5087sup1.cif

Structure factors: contains datablock(s) I. DOI: 10.1107/S1600536813030055/rz5087Isup2.hkl

Additional supplementary materials:  crystallographic information; 3D view; checkCIF report

## Figures and Tables

**Table 1:** Hydrogen-bond geometry (Å, °)

*D*—H⋯*A*	*D*—H	H⋯*A*	*D*⋯*A*	*D*—H⋯*A*
N1—H1⋯O7^i^	0.86	2.01	2.871 (6)	174
N3—H3⋯O6	0.86	1.96	2.619 (6)	133
O6—H6⋯O8^ii^	0.82	1.70	2.502 (9)	164

Symmetry codes: (i) 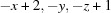; (ii) 
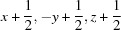
.

**Fig. 1.**
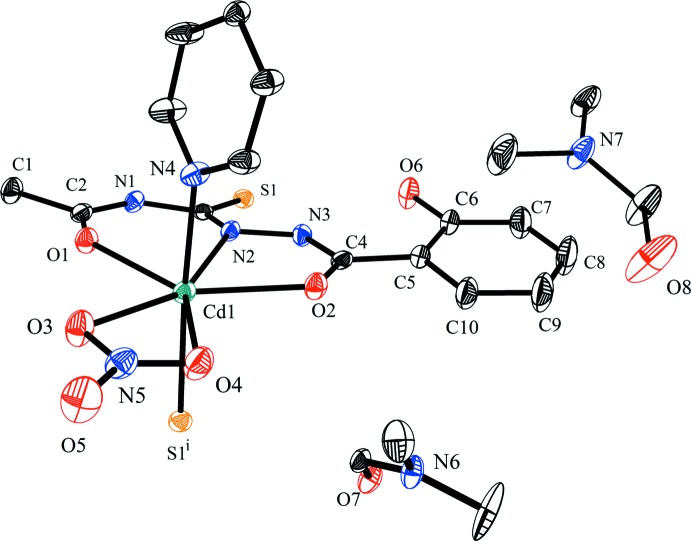
The asymmetric unit of the title complex with 30% probability displacement ellipsoids. H atoms are omitted for clarity. Symmetry code: (i) -x+2, -y, -z+1.

**Fig. 2.**
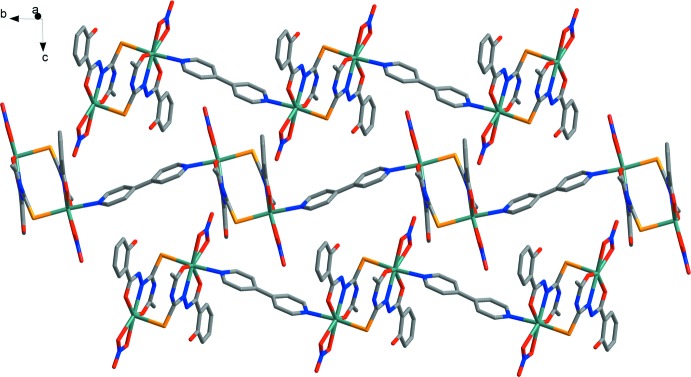
A view of the one-dimensional chains in the title compound. For clarity, H atoms and DMF molecules are omitted.

**Fig. 3.**
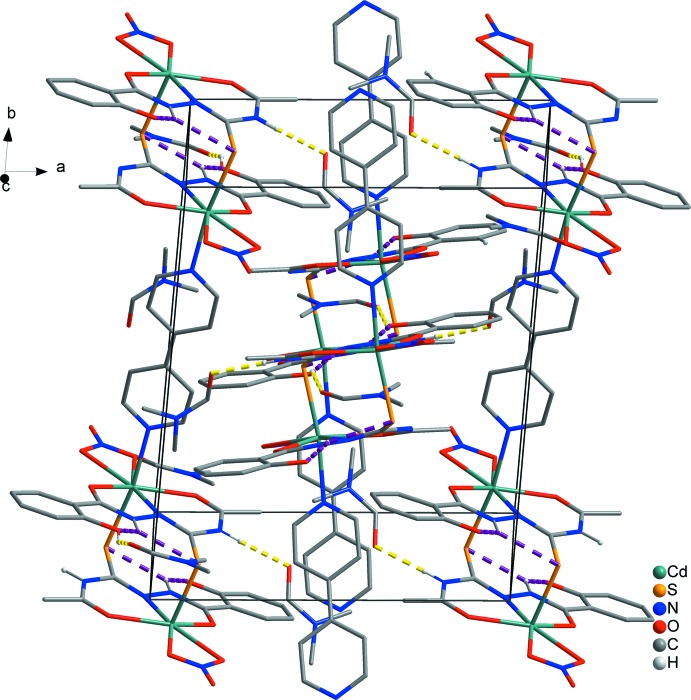
A packing diagram for title complex, showing intermolecular (yellow dashed lines) and intramolecular hydrogen bonds (purple dashed lines). H atoms not involved in hydrogen bonding are omitted.

## supplementary crystallographic information

### 1. Comment

Thiosemicarbazones complexes have received considerable attentions in the past decades due to their interesting biological activities, including antibacterial, antimalarial, antiviral and antitumor activities (Quiroga & Ranninger, 2004; Kasuga *et al.*, 2003;). In order to figure out their structure-property relationship, a great number of metal complexes based on thiosemicarbazone derivatives, particularly the 1,4-disubstituted ones have been prepared and their biological activities were investigated systematically (Floquet *et al.*, 2009; Hassanien *et al.*, 2008; Latheef *et al.*, 2006; Babb *et al.*, 2003). In this paper, we report the crystal structure of the title one-dimensional coordination polymer based on diacylthiosemicarbazone.

The asymmetric unit of the title complex consists of one cadmium(II) cation, one *N*-(2-(2-hydroxybenzoyl)carbamothioyl)acetamide ligand, one half of a 4,4'-bipyridine, one coordinated nitrate anion and two dimethylformamide molecules of crystallization (Fig. 1). In the structure, each Cd center adopts a distorted pentagonal bipyramidal coordination geometry with the equatorial plane formed two O atoms and one N atom from the thiosemicarbazide ligand and two O atoms from the bidentate nitrate anion. These five atoms (O1, N2, O2, O4, O3) and the metal center are almost coplanar (maximum deviation from the least-squares plane is 0.6560 (3) Å for the O3 atom). The Cd1—O3 and Cd1—O4 bond lengths involving the nitrate anion are remarkably different (2.387 (4) and 2.553 (5) Å, respectively). The axial positions are occupied by one N atom from 4,4'-bipyridine and one S atom from a neighbouring thiosemicarbazide ligand. The Cd1—N4 bond distance is 2.360 (5) Å, indicating the strong coordination between Cd and 4,4'-bipyridine, while the Cd1—S1^i^ [symmetry code: (i) -x+2, -y, -z+1] bond length is 2.628 (5) Å, which is slightly shorter than that previously reported (2.7364 (8) Å) for a Cd complex with thiosemicarbazones (Wang *et al.*, 2010). Due to the axial coordination of S atoms, two neighbouring cadmium(II) cations are interconnected to generate a centrosymmetric binuclear unit with a Cd···Cd separation of 5.6889 (12) Å (Fig. 2). Along the *b* axis, such binuclear units are further bridged by 4,4'-bipyridine linkers to form a one-dimensional zigzag coordination polymer. In the structure of the title complex, N—H···O and O—H···O hydrogen bonds (Table 1) play an important role in stabilizing the packing (Fig. 3).

### 2. Experimental

*N*-(2-(2-Hydroxybenzoyl)hydrazinecarbonothioyl)acetamide (H_3_L) was prepared according to the literature method (Wang *et al.*, 2000). H_3_L (0.0251 g, 0.10 mmol) and cadmium nitrate tetrahydrate (0.0472 g, 0.20 mmol) were dissolved in a mixed solvent of methanol and dimethylformamide (12 ml, 5:1 *v*/*v*). 4,4'-Bipyridine (0.0102 g 0.05 mmol) was added and the solution was stirred for 4 h at room temperature. The resulting white suspension was filltered and the filtrate allowed to evaporate in air at room temperature. Colourless crystals of the title compound were separated from the filtrate after 10 days.

### 3. Refinement

All C- and N-bound H atoms were placed in idealized positions using the riding-model approximation, with C—H = 0.93-0.96 Å, N—H = 0.86 Å, O—H = 0.86 Å, and with *U*_iso_(H) = 1.5*U*_eq_(C) for methyl groups and 1.2*U*_eq_(C, N, O) otherwise. In the last cycles of refinement, three outliers (-4 6 8, -3 5 6, 1 2 0) were omitted.

### Crystal data

**Table:**

[Cd_2_(C_10_H_10_N_3_O_3_S)_2_(C_10_H_8_N_2_)(NO_3_)_2_]·4C_3_H_7_NO	*F*(000) = 1324
*M**_r_* = 1301.93	*D*_x_ = 1.521 Mg m^−^^3^
Monoclinic, *P*2_1_/*n*	Mo *K*α radiation, λ = 0.71073 Å
Hall symbol: -P 2yn	Cell parameters from 10007 reflections
*a* = 13.831 (3) Å	θ = 3.0–27.5°
*b* = 15.280 (3) Å	µ = 0.90 mm^−^^1^
*c* = 14.363 (3) Å	*T* = 293 K
β = 110.55 (3)°	Block, colourless
*V* = 2842.4 (12) Å^3^	0.30 × 0.18 × 0.08 mm
*Z* = 2	

### Data collection

**Table:**

Rigaku Saturn 724+ CCD diffractometer	4980 independent reflections
Radiation source: fine-focus sealed tube	4753 reflections with *I* > 2σ(*I*)
Graphite monochromator	*R*_int_ = 0.043
ω scans at fixed χ = 45°	θ_max_ = 25.0°, θ_min_ = 3.0°
Absorption correction: numerical (*CrystalClear*; Rigaku, 2007)	*h* = −16→16
*T*_min_ = 0.813, *T*_max_ = 0.946	*k* = −18→18
18197 measured reflections	*l* = −16→17

### Refinement

**Table:**

Refinement on *F*^2^	Primary atom site location: structure-invariant direct methods
Least-squares matrix: full	Secondary atom site location: difference Fourier map
*R*[*F*^2^ > 2σ(*F*^2^)] = 0.060	Hydrogen site location: inferred from neighbouring sites
*wR*(*F*^2^) = 0.141	H-atom parameters constrained
*S* = 1.28	*w* = 1/[σ^2^(*F*_o_^2^) + (0.0474*P*)^2^ + 4.0842*P*] where *P* = (*F*_o_^2^ + 2*F*_c_^2^)/3
4980 reflections	(Δ/σ)_max_ < 0.001
349 parameters	Δρ_max_ = 0.70 e Å^−^^3^
0 restraints	Δρ_min_ = −0.63 e Å^−^^3^

### Special details

**Table:**

Geometry. All e.s.d.'s (except the e.s.d. in the dihedral angle between two l.s. planes) are estimated using the full covariance matrix. The cell e.s.d.'s are taken into account individually in the estimation of e.s.d.'s in distances, angles and torsion angles; correlations between e.s.d.'s in cell parameters are only used when they are defined by crystal symmetry. An approximate (isotropic) treatment of cell e.s.d.'s is used for estimating e.s.d.'s involving l.s. planes.
Refinement. Refinement of *F*^2^ against ALL reflections. The weighted *R*-factor *wR* and goodness of fit *S* are based on *F*^2^, conventional *R*-factors *R* are based on *F*, with *F* set to zero for negative *F*^2^. The threshold expression of *F*^2^ > σ(*F*^2^) is used only for calculating *R*-factors(gt) *etc*. and is not relevant to the choice of reflections for refinement. *R*-factors based on *F*^2^ are statistically about twice as large as those based on *F*, and *R*- factors based on ALL data will be even larger.

### Fractional atomic coordinates and isotropic or equivalent isotropic displacement parameters (Å^2^)

**Table:**

	*x*	*y*	*z*	*U*_iso_*/*U*_eq_	
Cd1	0.93079 (3)	0.13619 (3)	0.35688 (3)	0.05085 (16)	
N4	0.9607 (3)	0.2863 (3)	0.3953 (4)	0.0603 (11)	
O2	0.8129 (3)	0.1541 (3)	0.4400 (3)	0.0618 (10)	
C4	0.8420 (4)	0.1339 (3)	0.5294 (4)	0.0520 (12)	
N2	1.0120 (3)	0.1051 (3)	0.5329 (3)	0.0482 (10)	
C6	0.7984 (5)	0.1324 (4)	0.6899 (5)	0.0704 (17)	
O4	0.7810 (3)	0.1911 (3)	0.2065 (3)	0.0824 (13)	
C5	0.7705 (4)	0.1409 (4)	0.5870 (5)	0.0631 (15)	
O6	0.8984 (3)	0.1126 (4)	0.7441 (3)	0.0897 (15)	
H6	0.9043	0.1049	0.8024	0.108*	
O5	0.8031 (4)	0.2180 (5)	0.0673 (4)	0.118 (2)	
N5	0.8361 (4)	0.1955 (4)	0.1550 (4)	0.0747 (14)	
O3	0.9307 (3)	0.1752 (4)	0.1959 (3)	0.0837 (13)	
C7	0.7240 (6)	0.1425 (6)	0.7354 (6)	0.097 (2)	
H7	0.7435	0.1375	0.8041	0.116*	
C10	0.6671 (5)	0.1583 (6)	0.5319 (6)	0.094 (2)	
H10	0.6465	0.1641	0.4632	0.113*	
C8	0.6238 (7)	0.1596 (8)	0.6796 (8)	0.129 (4)	
H8	0.5750	0.1662	0.7101	0.154*	
C9	0.5949 (6)	0.1669 (8)	0.5777 (8)	0.131 (4)	
H9	0.5262	0.1778	0.5395	0.157*	
O1	1.1075 (3)	0.1222 (3)	0.3888 (3)	0.0616 (10)	
N1	1.1819 (3)	0.0725 (3)	0.5489 (3)	0.0519 (10)	
H1	1.2402	0.0541	0.5895	0.062*	
N3	0.9386 (3)	0.1056 (3)	0.5792 (3)	0.0510 (10)	
H3	0.9551	0.0879	0.6396	0.061*	
C3	1.1007 (4)	0.0711 (3)	0.5871 (4)	0.0470 (11)	
C13	0.9920 (4)	0.4559 (3)	0.4777 (4)	0.0540 (12)	
C2	1.1834 (4)	0.0978 (4)	0.4592 (4)	0.0542 (13)	
N6	0.5321 (4)	0.1122 (4)	0.2567 (5)	0.0908 (18)	
C15	0.8837 (5)	0.3441 (4)	0.3760 (5)	0.0714 (17)	
H15	0.8182	0.3272	0.3343	0.086*	
C14	0.8962 (4)	0.4273 (4)	0.4146 (5)	0.0675 (16)	
H14	0.8398	0.4649	0.3983	0.081*	
C16	0.6130 (4)	0.0601 (5)	0.2860 (5)	0.0717 (17)	
H16	0.6719	0.0790	0.2747	0.086*	
C11	1.0539 (5)	0.3136 (4)	0.4533 (6)	0.089 (2)	
H11	1.1093	0.2752	0.4667	0.107*	
C12	1.0716 (5)	0.3966 (4)	0.4944 (6)	0.093 (2)	
H12	1.1383	0.4126	0.5340	0.111*	
C17	0.5353 (8)	0.1979 (6)	0.2137 (8)	0.126 (3)	
H17A	0.6029	0.2080	0.2114	0.189*	
H17B	0.5198	0.2422	0.2538	0.189*	
H17C	0.4852	0.2002	0.1476	0.189*	
C18	0.4398 (7)	0.0860 (10)	0.2729 (12)	0.218 (8)	
H18A	0.4575	0.0504	0.3316	0.327*	
H18B	0.3970	0.0529	0.2167	0.327*	
H18C	0.4030	0.1369	0.2811	0.327*	
O7	0.6180 (3)	−0.0114 (4)	0.3271 (4)	0.0868 (14)	
C1	1.2880 (4)	0.0956 (5)	0.4484 (5)	0.0683 (16)	
H1A	1.2857	0.0572	0.3948	0.103*	
H1B	1.3385	0.0748	0.5091	0.103*	
H1C	1.3062	0.1535	0.4344	0.103*	
S1	1.12907 (10)	0.02354 (9)	0.70412 (10)	0.0547 (3)	
N7	0.5920 (7)	0.3996 (9)	0.5419 (6)	0.167 (4)	
C20	0.6425 (12)	0.4109 (13)	0.4690 (11)	0.249 (9)	
H20A	0.5981	0.3896	0.4055	0.374*	
H20B	0.7062	0.3788	0.4899	0.374*	
H20C	0.6565	0.4719	0.4637	0.374*	
C21	0.6531 (8)	0.3997 (10)	0.6491 (8)	0.169 (5)	
H21A	0.6078	0.3928	0.6862	0.253*	
H21B	0.6897	0.4541	0.6668	0.253*	
H21C	0.7016	0.3522	0.6641	0.253*	
O8	0.4333 (8)	0.3831 (12)	0.4272 (6)	0.334 (11)	
C19	0.4931 (10)	0.3835 (13)	0.5125 (8)	0.235 (10)	
H19	0.4658	0.3713	0.5617	0.282*	

### Atomic displacement parameters (Å^2^)

**Table:**

	*U*^11^	*U*^22^	*U*^33^	*U*^12^	*U*^13^	*U*^23^
Cd1	0.0399 (2)	0.0541 (3)	0.0550 (3)	0.00047 (16)	0.01226 (17)	−0.00090 (17)
N4	0.050 (3)	0.049 (3)	0.074 (3)	−0.001 (2)	0.012 (2)	−0.001 (2)
O2	0.044 (2)	0.076 (3)	0.063 (2)	0.0082 (18)	0.0158 (18)	0.003 (2)
C4	0.038 (3)	0.051 (3)	0.065 (3)	−0.001 (2)	0.014 (2)	−0.011 (3)
N2	0.036 (2)	0.048 (2)	0.062 (3)	−0.0030 (18)	0.0188 (19)	−0.003 (2)
C6	0.054 (3)	0.086 (4)	0.080 (4)	0.000 (3)	0.034 (3)	−0.010 (3)
O4	0.057 (2)	0.107 (4)	0.083 (3)	−0.004 (2)	0.024 (2)	0.004 (3)
C5	0.049 (3)	0.068 (4)	0.076 (4)	−0.003 (3)	0.026 (3)	−0.012 (3)
O6	0.057 (3)	0.145 (5)	0.070 (3)	0.013 (3)	0.027 (2)	0.010 (3)
O5	0.082 (3)	0.181 (6)	0.073 (3)	0.012 (4)	0.007 (3)	0.047 (4)
N5	0.064 (3)	0.086 (4)	0.064 (3)	0.000 (3)	0.011 (3)	0.010 (3)
O3	0.054 (3)	0.126 (4)	0.068 (3)	0.013 (3)	0.016 (2)	0.016 (3)
C7	0.082 (5)	0.138 (7)	0.089 (5)	0.008 (5)	0.052 (4)	−0.006 (5)
C10	0.047 (4)	0.147 (7)	0.091 (5)	0.008 (4)	0.026 (3)	−0.016 (5)
C8	0.074 (6)	0.217 (12)	0.118 (7)	0.014 (6)	0.062 (5)	−0.008 (7)
C9	0.049 (4)	0.228 (12)	0.120 (7)	0.025 (6)	0.034 (4)	−0.019 (8)
O1	0.043 (2)	0.081 (3)	0.059 (2)	0.0057 (18)	0.0148 (18)	0.0052 (19)
N1	0.033 (2)	0.056 (3)	0.064 (3)	0.0051 (18)	0.0145 (19)	0.001 (2)
N3	0.042 (2)	0.058 (3)	0.054 (2)	0.0056 (19)	0.0178 (19)	−0.002 (2)
C3	0.038 (2)	0.041 (3)	0.058 (3)	−0.001 (2)	0.013 (2)	−0.009 (2)
C13	0.042 (3)	0.051 (3)	0.063 (3)	−0.001 (2)	0.011 (2)	0.002 (2)
C2	0.041 (3)	0.052 (3)	0.068 (3)	−0.005 (2)	0.017 (3)	−0.007 (3)
N6	0.056 (3)	0.108 (5)	0.110 (5)	0.027 (3)	0.032 (3)	0.027 (4)
C15	0.049 (3)	0.061 (4)	0.086 (4)	0.003 (3)	0.002 (3)	−0.010 (3)
C14	0.048 (3)	0.055 (3)	0.086 (4)	0.006 (3)	0.007 (3)	−0.004 (3)
C16	0.039 (3)	0.102 (5)	0.069 (4)	0.004 (3)	0.012 (3)	−0.005 (4)
C11	0.051 (4)	0.060 (4)	0.133 (6)	0.007 (3)	0.003 (4)	−0.024 (4)
C12	0.042 (3)	0.064 (4)	0.144 (7)	−0.002 (3)	−0.002 (4)	−0.031 (4)
C17	0.126 (8)	0.112 (7)	0.150 (9)	0.042 (6)	0.061 (7)	0.028 (7)
C18	0.066 (6)	0.262 (16)	0.35 (2)	0.055 (8)	0.103 (9)	0.148 (15)
O7	0.056 (3)	0.102 (4)	0.096 (3)	0.014 (2)	0.019 (2)	0.022 (3)
C1	0.042 (3)	0.087 (4)	0.081 (4)	0.000 (3)	0.028 (3)	0.000 (3)
S1	0.0464 (7)	0.0602 (8)	0.0516 (7)	0.0013 (6)	0.0098 (6)	−0.0056 (6)
N7	0.105 (6)	0.306 (14)	0.098 (6)	−0.052 (8)	0.043 (5)	0.008 (7)
C20	0.192 (15)	0.40 (3)	0.210 (15)	−0.070 (17)	0.140 (13)	0.001 (17)
C21	0.094 (7)	0.279 (16)	0.115 (8)	−0.040 (9)	0.012 (6)	−0.018 (9)
O8	0.182 (9)	0.72 (3)	0.071 (5)	−0.177 (13)	0.015 (5)	0.056 (9)
C19	0.119 (9)	0.51 (3)	0.070 (6)	−0.087 (13)	0.028 (6)	0.031 (11)

### Geometric parameters (Å, º)

**Table:**

Cd1—O1	2.332 (4)	C13—C14	1.388 (7)
Cd1—O2	2.350 (4)	C13—C13^ii^	1.476 (10)
Cd1—N4	2.362 (5)	C2—C1	1.509 (7)
Cd1—O3	2.387 (4)	N6—C16	1.317 (8)
Cd1—N2	2.426 (4)	N6—C18	1.433 (10)
Cd1—O4	2.553 (5)	N6—C17	1.456 (10)
Cd1—S1^i^	2.6270 (15)	C15—C14	1.373 (8)
N4—C11	1.332 (7)	C15—H15	0.9300
N4—C15	1.336 (7)	C14—H14	0.9300
O2—C4	1.242 (7)	C16—O7	1.232 (8)
C4—N3	1.346 (6)	C16—H16	0.9300
C4—C5	1.499 (8)	C11—C12	1.384 (9)
N2—C3	1.307 (6)	C11—H11	0.9300
N2—N3	1.396 (5)	C12—H12	0.9300
C6—O6	1.361 (8)	C17—H17A	0.9600
C6—C5	1.397 (9)	C17—H17B	0.9600
C6—C7	1.408 (9)	C17—H17C	0.9600
O4—N5	1.237 (6)	C18—H18A	0.9600
C5—C10	1.395 (9)	C18—H18B	0.9600
O6—H6	0.8200	C18—H18C	0.9600
O5—N5	1.229 (7)	C1—H1A	0.9600
N5—O3	1.270 (6)	C1—H1B	0.9600
C7—C8	1.361 (12)	C1—H1C	0.9600
C7—H7	0.9300	S1—Cd1^i^	2.6270 (15)
C10—C9	1.382 (10)	N7—C19	1.305 (14)
C10—H10	0.9300	N7—C20	1.458 (13)
C8—C9	1.380 (12)	N7—C21	1.474 (12)
C8—H8	0.9300	C20—H20A	0.9600
C9—H9	0.9300	C20—H20B	0.9600
O1—C2	1.232 (6)	C20—H20C	0.9600
N1—C2	1.352 (7)	C21—H21A	0.9600
N1—C3	1.412 (6)	C21—H21B	0.9600
N1—H1	0.8600	C21—H21C	0.9600
N3—H3	0.8600	O8—C19	1.214 (13)
C3—S1	1.745 (5)	C19—H19	0.9300
C13—C12	1.381 (8)		
			
O1—Cd1—O2	140.93 (13)	N2—C3—S1	125.9 (4)
O1—Cd1—N4	87.55 (15)	N1—C3—S1	116.1 (3)
O2—Cd1—N4	82.21 (15)	C12—C13—C14	115.3 (5)
O1—Cd1—O3	81.83 (14)	C12—C13—C13^ii^	122.4 (6)
O2—Cd1—O3	134.28 (14)	C14—C13—C13^ii^	122.3 (6)
N4—Cd1—O3	85.65 (18)	O1—C2—N1	125.1 (5)
O1—Cd1—N2	72.96 (13)	O1—C2—C1	119.6 (5)
O2—Cd1—N2	69.13 (13)	N1—C2—C1	115.3 (5)
N4—Cd1—N2	88.16 (15)	C16—N6—C18	119.0 (8)
O3—Cd1—N2	154.27 (14)	C16—N6—C17	122.1 (7)
O1—Cd1—O4	132.81 (14)	C18—N6—C17	118.8 (7)
O2—Cd1—O4	83.67 (14)	N4—C15—C14	123.3 (5)
N4—Cd1—O4	84.38 (16)	N4—C15—H15	118.3
O3—Cd1—O4	51.27 (14)	C14—C15—H15	118.3
N2—Cd1—O4	152.53 (14)	C15—C14—C13	120.8 (5)
O1—Cd1—S1^i^	99.58 (10)	C15—C14—H14	119.6
O2—Cd1—S1^i^	94.80 (10)	C13—C14—H14	119.6
N4—Cd1—S1^i^	171.58 (11)	O7—C16—N6	126.0 (6)
O3—Cd1—S1^i^	90.91 (14)	O7—C16—H16	117.0
N2—Cd1—S1^i^	98.16 (10)	N6—C16—H16	117.0
O4—Cd1—S1^i^	87.47 (12)	N4—C11—C12	122.8 (6)
C11—N4—C15	116.7 (5)	N4—C11—H11	118.6
C11—N4—Cd1	120.1 (4)	C12—C11—H11	118.6
C15—N4—Cd1	122.1 (4)	C13—C12—C11	121.1 (5)
C4—O2—Cd1	117.7 (3)	C13—C12—H12	119.5
O2—C4—N3	122.0 (5)	C11—C12—H12	119.5
O2—C4—C5	121.0 (5)	N6—C17—H17A	109.5
N3—C4—C5	117.0 (5)	N6—C17—H17B	109.5
C3—N2—N3	114.0 (4)	H17A—C17—H17B	109.5
C3—N2—Cd1	133.8 (3)	N6—C17—H17C	109.5
N3—N2—Cd1	110.3 (3)	H17A—C17—H17C	109.5
O6—C6—C5	118.2 (5)	H17B—C17—H17C	109.5
O6—C6—C7	121.3 (7)	N6—C18—H18A	109.5
C5—C6—C7	120.4 (6)	N6—C18—H18B	109.5
N5—O4—Cd1	92.1 (3)	H18A—C18—H18B	109.5
C10—C5—C6	117.7 (6)	N6—C18—H18C	109.5
C10—C5—C4	116.4 (6)	H18A—C18—H18C	109.5
C6—C5—C4	125.9 (5)	H18B—C18—H18C	109.5
C6—O6—H6	109.5	C2—C1—H1A	109.5
O5—N5—O4	123.0 (6)	C2—C1—H1B	109.5
O5—N5—O3	119.6 (6)	H1A—C1—H1B	109.5
O4—N5—O3	117.4 (5)	C2—C1—H1C	109.5
N5—O3—Cd1	99.2 (3)	H1A—C1—H1C	109.5
C8—C7—C6	120.5 (8)	H1B—C1—H1C	109.5
C8—C7—H7	119.8	C3—S1—Cd1^i^	97.32 (16)
C6—C7—H7	119.8	C19—N7—C20	120.1 (10)
C9—C10—C5	121.1 (8)	C19—N7—C21	119.3 (9)
C9—C10—H10	119.4	C20—N7—C21	120.4 (10)
C5—C10—H10	119.4	N7—C20—H20A	109.5
C7—C8—C9	119.8 (7)	N7—C20—H20B	109.5
C7—C8—H8	120.1	H20A—C20—H20B	109.5
C9—C8—H8	120.1	N7—C20—H20C	109.5
C8—C9—C10	120.6 (8)	H20A—C20—H20C	109.5
C8—C9—H9	119.7	H20B—C20—H20C	109.5
C10—C9—H9	119.7	N7—C21—H21A	109.5
C2—O1—Cd1	136.2 (4)	N7—C21—H21B	109.5
C2—N1—C3	130.9 (4)	H21A—C21—H21B	109.5
C2—N1—H1	114.6	N7—C21—H21C	109.5
C3—N1—H1	114.6	H21A—C21—H21C	109.5
C4—N3—N2	120.1 (4)	H21B—C21—H21C	109.5
C4—N3—H3	119.9	O8—C19—N7	126.4 (11)
N2—N3—H3	119.9	O8—C19—H19	116.8
N2—C3—N1	118.1 (5)	N7—C19—H19	116.8

Symmetry codes: (i) −*x*+2, −*y*, −*z*+1; (ii) −*x*+2, −*y*+1, −*z*+1.

### Hydrogen-bond geometry (Å, º)

**Table:**

*D*—H···*A*	*D*—H	H···*A*	*D*···*A*	*D*—H···*A*
N1—H1···O7^i^	0.86	2.01	2.871 (6)	174
N3—H3···O6	0.86	1.96	2.619 (6)	133
N3—H3···S1	0.86	2.46	2.901 (4)	113
O6—H6···O8^iii^	0.82	1.70	2.502 (9)	164

Symmetry codes: (i) −*x*+2, −*y*, −*z*+1; (iii) *x*+1/2, −*y*+1/2, *z*+1/2.

## References

[bb1] Babb, J. E. V., Burrows, A. D., Harrington, R. W. & Mahon, M. F. (2003). *Polyhedron*, **22**, 673–686.

[bb2] Floquet, S., Muñoz, M. C., Guillot, R., Riviére, E., Blain, G., Réal, J.-A. & Boillot, M.-L. (2009). *Inorg. Chim. Acta*, **362**, 56–64.

[bb3] Hassanien, M. M., Gabr, I. M., Abdel-Rhman, M. H. & El-Asmy, A. A. (2008). *Spectrochim. Acta Part A*, **71**, 73–79. 10.1016/j.saa.2007.11.009 18164649

[bb4] Kasuga, N. C., Sekino, K., Ishikawa, M., Honda, A., Yokoyama, M., Nakano, S., Shimada, N., Koumo, C. & Nomiya, K. (2003). *J. Inorg. Biochem.* **96**, 298–310. 10.1016/s0162-0134(03)00156-9 12888265

[bb5] Ke, Y.-Z., Zheng, L.-F., Luo, J.-H., Huang, X.-H. & Huang, C.-C. (2007). *Acta Cryst.* C**63**, m343–m345. 10.1107/S0108270107027631 17675678

[bb6] Latheef, L., Manoj, E. & Prathapachandra Kurup, M. R. (2006). *Acta Cryst.* C**62**, o16–o18. 10.1107/S010827010503564X 16397330

[bb7] Liu, J.-J., Liao, J.-Z., Li, Z.-Y., Wang, Y. & Huang, C.-C. (2013). *Acta Cryst.* C**69**, 613–615. 10.1107/S0108270113012547 23744379

[bb8] Quiroga, A. G. & Ranninger, C. N. (2004). *Coord. Chem. Rev.* **248**, 119–133.

[bb9] Rigaku (2007). *CrystalClear.* Rigaku Corporation, Tokyo, Japan.

[bb10] Sheldrick, G. M. (2008). *Acta Cryst.* A**64**, 112–122. 10.1107/S0108767307043930 18156677

[bb11] Wang, X., Li, Z., Da, Y. & Chen, J. (2000). *Synth. Commun.* **30**, 3405–3414.

[bb12] Wang, Y.-B., Pan, T.-H., Liang, Q., Liu, D.-S. & Huang, C.-C. (2010). *Acta Cryst.* C**66**, m127–m129. 10.1107/S0108270110011455 20442501

